# RBX1 prompts degradation of EXO1 to limit the homologous recombination pathway of DNA double-strand break repair in G1 phase

**DOI:** 10.1038/s41418-019-0424-4

**Published:** 2019-09-27

**Authors:** Ying Xie, Yi-Ke Liu, Zong-Pei Guo, Hua Guan, Xiao-Dan Liu, Da-Fei Xie, Yi-Guo Jiang, Teng Ma, Ping-Kun Zhou

**Affiliations:** 1 Department of Radiation Toxicology and Oncology, Beijing Institute of Radiation Medicine, 100850 Beijing, China; 2 Key Laboratory of Molecular Epidemiology of Hunan Province, School of Medicine, Hunan Normal University, 410013 Changsha, China; 3 Institute for Chemical Carcinogenesis, State Key Laboratory of Respiratory Disease, Guangzhou Medical University, 511436 Guangzhou, China; 4 Department of Cellular and Molecular Biology, Beijing Chest Hospital, Capital Medical University/Beijing Tuberculosis and Thoracic Tumor Research Institute, 101149 Beijing, China

**Keywords:** DNA, Protein quality control

## Abstract

End resection of DNA double-strand breaks (DSBs) to form 3′ single-strand DNA (ssDNA) is critical to initiate the homologous recombination (HR) pathway of DSB repair. HR pathway is strictly limited in the G1-phase cells because of lack of homologous DNA as the templates. Exonuclease 1 (EXO1) is the key molecule responsible for 3′ ssDNA formation of DSB end resection. We revealed that EXO1 is inactivated in G1-phase cells via ubiquitination-mediated degradation, resulting from an elevated expression level of RING-box protein 1 (RBX1) in G1 phase. The increased RBX1 significantly prompted the neddylation of Cullin1 and contributed to the G1 phase-specific degradation of EXO1. Knockdown of RBX1 remarkedly attenuated the degradation of EXO1 and increased the end resection and HR activity in γ-irradiated G1-phase cells, as demonstrated by the increased formation of RPA32, BrdU, and RAD51 foci. And EXO1 depletion mitigated DNA repair defects due to RBX1 reduction. Moreover, increased autophosphorylation of DNA-PKcs at S2056 was found to be responsible for the higher expression level of the RBX1 in the G1 phase. Inactivation of DNA-PKcs decreased RBX1 expression, and simultaneously increased EXO1 expression and DSB end resection in G1-phase cells. This study demonstrates a new mechanism for restraining the HR pathway of DNA DSB repair in G1 phase via RBX1-prompted inactivation of EXO1.

## Introduction

DNA double-strand breaks (DSBs) are among the most lethal types of DNA damage. Failure to properly repair DSBs leads to chromosomal aberrations, an overall increase in genomic instability and cell death [1–5]. There are two major mechanistic pathways in mammalian cells to repair DSBs: error-prone non-homologous end-joining (NHEJ) or error-free homologous recombination (HR) [6, 7]. The HR pathway repairs DSBs using a homologous DNA sequence as the template, while the NHEJ pathway directly ligases the two unresected ends in the absence of template strands [8]. Obviously, HR repair is restricted to late S-phase and G2-phase cells, while NHEJ repair can take place at any time during the cell cycle, especially in G1 phase [9]. Therefore, in G1 phase, NHEJ is preferably adapted to repair DSBs, while HR activity is tightly restrained due to the lack of necessary templates for homologous DNA. It is still an important question how to guarantee the proper DSB repair pathway choice during the cell cycle.

The HR pathway is initiated by the end resection of DSBs to generate 3′ single-stranded DNA (ssDNA) overhangs. Although resection-dependent c-NHEJ was reported in G1 cells [5], it is well known that once DNA end resection has initiated, the NHEJ pathway can no longer be efficiently used for DSB re-joining [2]. DNA end resection is initiated by the Mre11‐Rad50‐Xrs2 (MRX) complex to generate short 3′ overhangs [10, 11]. The nucleases exonuclease 1 (EXO1) and DNA2 subsequently function to extend these short overhangs into long sequences of ssDNA, leading to RPA32 displacement, and RAD51 nucleofilament formation in the HR pathway [12]. Unlike Mre11 or CtIP, depletion of EXO1 causes a severe resection defect based on the reduced phosphorylation of RPA32 at serine 4/8, suggesting that EXO1 plays a pivotal role in end resection and HR repair [13, 14]. Despite its critical role in inducing the overall DNA damage response, it is important to understand whether and how EXO1 nuclease plays a role in restraining HR activity in G1-phase cells to guarantee the proper choice of the DSB repair pathway.

EXO1 was reported to be rapidly degraded via a ubiquitin-mediated proteasome pathway in response to DNA damage [15]. Several studies have suggested that EXO1 ubiquitination is mediated by the Skp1-Cullin1-F-box (SCF) ubiquitin ligase in a phosphorylation-dependent manner [15, 16]. However, considering that EXO1 is phosphorylated and executes end resection function in the G2 phase, it is still unclear how phosphorylation of EXO1 facilitates its ubiquitination degradation. In addition, it is reasonable that in G1-phase cells, the extended end resection of DSBs should be tightly restrained, as it is a large obstacle for NHEJ and deleterious for the survival of cells harboring DSBs. The status of EXO1 activity in G1 cells and whether it is a key regulatory component of DSB repair pathway determination for G1 cells is unclear. Therefore, we investigated cell cycle-dependent alterations and the mechanism of EXO1 activity/degradation and its significance in the regulation of DSB repair pathway choice in G1-phase cells.

## Material and methods

### Cell culture and treatment

HeLa, MCF7, A549, and U2OS cells were grown in DMEM medium supplemented with 10% fetal bovine serum and 1% penicillin/streptomycin. A cobalt‐60γ‐ray source (Beijing Institute of Radiation Medicine) was used to irradiate the cells at a dose rate of 88.14 cGy/min at room temperature. Nu7026 (Selleck), MLN4924 (MedChem Express), MG132 (Merck), thymidine (TDR; Sigma-Aldrich), and cycloheximide (CHX; Sigma-Aldrich) were used at final concentrations of 10 μM, 5 μM, 10 μM, 2 mM, and 100 μg/ml, respectively.

### Plasmid DNA and siRNA transfections

Plasmid DNA and siRNA transfections were performed using Lipofectamine 2000 (Invitrogen) following the manufacturer’s instructions. FLAG-tagged HA-Ub and SFB-Ub were used for co-immunoprecipitation experiments to detect ubiquitination levels of EXO1. The siRNA sequences targeting human RBX1 were CUGGGAUAUUGUGGUUGAUTT and GAAGCGCUUUGAAGUGAAATT. The siRNA sequences targeting human Cullin1 were GCUCUACACUCAUGUUUAUTT and GAACCCAGUUACUGAAUAUTT. The siRNA sequence targeting human EXO1 was CCAAUCUUCUUAAGGGAAATT. All of the siRNA duplexes were obtained from Genechem (Shanghai, China).

### Antibodies

All antibodies were purchased commercially: anti-p-DNA-PKcs-S2056 (Abcam; ab174576), anti-EXO1 (Abcam; ab95012), anti-ubiquitinated protein antibody (Millipore; 2558438), anti-phospho-(Ser/Thr)(Abcam; ab17464), anti-NEDD8 (Abcam; ab81264), anti-RBX1 (CST; 4397), anti-Cullin1 (CST; 4995), anti-Ub (Millipore; 2956794 A), and anti-β-actin (Abcam; ab8226). Secondary antibodies were HRP-conjugated anti-rabbit IgG (H + L) or HRP-conjugated anti-mouse IgG (H + L) purchased from Zhongshan Golden Bridge Biotechnology. For the immunofluorescence staining assay, anti-CENP-F (Abnova; MAB12563), anti-RBX1 (Abcam; ab2977), anti-RAD51 (Millipore; 24912220), and anti-ץH2AX (Millipore; 2714539) antibodies were used. Fluorescence-conjugated anti-mouse immunoglobulin G (IgG) and anti-rabbit immunoglobulin G (IgG) were purchased from Molecular Probes (Alexa Fluor 488 and 568).

### Cell synchronization and cell cycle analysis

HeLa cells were incubated with 2 mM thymidine for 17 h, released in fresh medium for 10 h, and then treated with thymidine for another 13 h. Cells were collected at different times after release for cell cycle analysis and western blotting. Cells were washed with pre-chilled PBS and stained with 10 μg/mL propidium iodide and 100 μg/mL RNase in PBS and then analysed by fluorescence-activated cell sorting.

### Western blotting and immunoprecipitation assays

Total cellular proteins were extracted with ice-cold NETN300 lysis buffer [20 mM Tris pH 8.0, 1 mM EDTA, 300 mM NaCl, 0.5% NP-40; protease inhibitor cocktail (Roche)] for 20 min and centrifuged at 12000 × *g* for 15 min at 4 °C. Protein detection by western blot analysis was performed following separation of whole-cell extracts (50 μg). For the immunoprecipitation assay, cell lysates were incubated with protein A/G agarose and primary antibody overnight. The agarose beads were then washed three times with lysis buffer and re-suspended in SDS-PAGE loading buffer for western blotting analysis using appropriate antibodies.

### Immunofluorescence staining assay

Cells cultured on glass coverslips were treated as indicated in the figure legends. After washing with PBS, cells were fixed in 4% paraformaldehyde for 15 min and permeabilized in 0.25% Triton X-100 solution for 30 min at room temperature. Cells were blocked with 1% BSA and incubated with primary antibody overnight. Subsequently, the samples were washed and incubated with secondary antibody for 60 min. DAPI staining was performed to visualize nuclear DNA. Coverslips were mounted onto glass slides and visualized using a Nikon ECLIPSE E800 fluorescence microscope.

### Detection of ssDNA by immunofluorescence

Cells on microscope slides were grown in 10 μM BrdU for at least 16 h, then were irradiated with 10 Gy. Cell were fixed in 4% paraformaldehyde for 15 min and permeabilized in 0.25% Triton X-100 solution for 30 min at room temperature. The coverslip rinsed in 2 M HCl at 37°C for 1 h and then were neutralized with 0.1 M sodium borate for 30 min. And cells were incubated with primary antibody overnight, and counterstained with secondary antibody and DAPI as described before.

### RT-PCR

Total RNA was isolated by Trizol reagent and reverse transcribed using ReverTra Ace qPCR RT Master Mix (Toyobo, FSQ-301). The following sense and antisense primer sequences were used: Cullin1-S, 5′- GCTGCTTTAAATGACCCCAA-3′; Cullin1-AS, 5′-TGTTGTTTATGAAGCGACCAC-3′; Skp1-S, 5′-AAGCGAACAGATGATATCCCT-3′; Skp1-AS, 5′-CCCCTTGATCATATTGGCAAC -3′; RBX1-S, 5′-CTGGCTCAAAACACGACAGG-3′; RBX1-AS, 5′-AGCATCCGTTCCAGAATCCAA-3′; EXO1-S, 5′-CTCAGCCATTCTTACTACGCTA-3′; EXO1-AS, 5′-AAGCCAGCAATATGTATCCAC-3′; β-actin-S, 5′-TGTCCACCTTCCAGCAGATGT-3′; β-actin-AS, 5′-CACCTTCACCGTTCCAGTTTT-3′. Human β-actin mRNA levels were used for normalization of SYBR-green real-time RT-PCR results.

### Colony formation assay

RBX1 was knocked down with siRNA in HeLa cells for 48 h. Next, the cells were re-seeded in a six-well plate and irradiated with 2 and 4 Gy. Then, the cells were cultured as normal in medium for 10 days. The colonies were stained with crystal violet and allowed to air dry at room temperature. The experiments were performed in triplicate, and the numbers of colonies containing more than 50 cells were microscopically counted to calculate the colony formation rate as the number of colonies/number of cells ×100%.

### Neutral comet assay (single cell gel electrophoresis assay)

The neutral comet assay was performed to detect DNA damage. HeLa cells were transfected with RBX1 siRNA for 48 h and then irradiated with 4 Gy and harvested at different times for the comet assay. Olive tail moments of comet images were determined using CASP software. For each experiment, 50 cells were scored from replicate slides (100 cells total), and the experiments were repeated three times.

### Statistical analysis

The results are expressed as the mean ± standard deviation and were calculated from quantitative data obtained from three replicate experiments. Statistical analysis was performed using one-way analysis of variance in SPSS v17.0 software. The significance of the differences between two groups were determined using LSD *t*-test. The *p*-values ≤ 0.05 were considered significant.

## Results

### Ubiquitination-mediated degradation of EXO1 is selectively activated in G1 phase

**Fig. 1:**
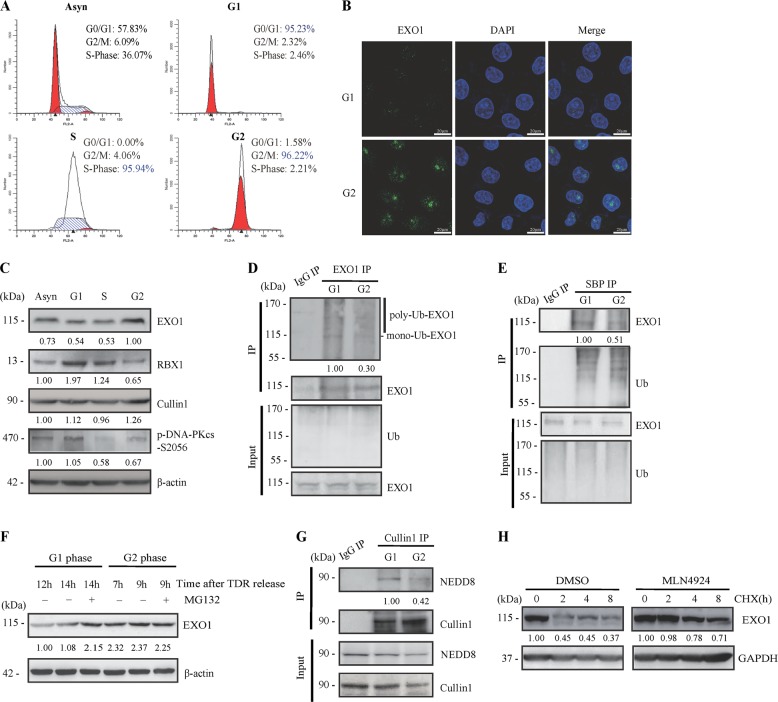
High-level ubiquitination mediated suppression of EXO1 in G1-phase cells. **a** HeLa cells were synchronized to different phases of the cell cycle by double-blockage of thymidine, and the cell phase distributions were monitored by flow cytometry analysis. **b** EXO1 protein was detected in the synchronized G1- and G2-phase HeLa cells by microscopic observation of immunofluorescent staining with anti-EXO1 antibody. Nuclei were stained with DAPI. **c** The expression levels of EXO1, RBX1, Cullin1, and phosphorylated DNA-PKcs-S2056 were assessed by western blotting analysis. Among them, phosphorylation of DNA-PKcs, is indicative of cells in different phase of cell cycle. **d**, **e** Ubiquitination of EXO1 protein was detected respectively in the synchronized G1- and G2-phase cells. After HeLa were transfected with HA-Ub and SBP-Ub plasmid for 48 h, the immunoprecipitation (IP) product obtained with the EXO1 antibody and S-bead agarose, were subjected to western blotting analysis with the anti-flag antibody and anti-EXO1 antibody separately. In order to achieve the same content of EXO1 for immunoprecipitation, when HA-Ub and SBP-Ub transfected cells were synchronized in G1- and G2-phase using a double thymidine block, cell were pre-treated with proteasome inhibitor MG132 for 2 h. **f** Hela were arrested at the G1/S boundary followed by a release into fresh media to allow cells to progress through the cell cycle. Cells were synchronized in G2 phase (7–9h) and G1 phase (12–20h) after TDR release. 10 μM MG132 was added in G1 and G2 cells for 2 h and EXO1 contents were detected. **g** Neddylation of Cullin1 protein was detected separately in the synchronized G1- and G2-phase cells. The immunoprecipitation (IP) product obtained with the Cullin1 antibody was subjected to western blotting analysis with anti-NEDD8 antibody. **h** The effect of neddylation on the stability of the EXO1 protein. Cells were pre-treated with the neddylation inhibitor MLN4924 or DMSO as a control for 8 h and then co-cultured with cycloheximide (CHX). EXO1 levels were assessed by western blotting analysis at the indicated times after CHX treatment

It has been well demonstrated that EXO1 functions in the end resection and HR pathway of DSB repair in the G2 phase and is associated with its phosphorylation by CDK1/2 [17, 18]. Theoretically, the function of EXO1 is stringently restrained in G1 phase, where the HR pathway does not work due to a lack of the homologous DNA template for repair. To explore the mechanism by which EXO1-dependent end resection is limited and guarantees the use of the NHEJ repair pathway in G1 phase, we initially examined the expression level of the EXO1 protein among different phases of the cell cycle. After cell cycle synchronization, we obtained more than 90% of cells synchronized in each phase (Fig. 1a). We found that the fluorescence intensity of EXO1 expression was lower in G1-phase cells in immunofluorescence staining assay (Fig. 1b). And we also observed that the level of EXO1 protein was markedly lower in the G1 phase than in G2 phases (Fig. 1c). To confirm the cell cycle-related differential expression of EXO1, we simultaneously measured the expression dynamics of phosphorylated DNA-dependent protein kinase catalytic subunit (DNA-PKcs)-S2056 in the cell cycle. The result showed a higher phosphorylated DNA-PKcs/S2056 in the G1 phase compared to the S and G2 phases (Fig. 1c), which was in accord with previous reports [19, 20]. We therefore assumed that ubiquitination-mediated degradation may contribute to the lower level of EXO1 in G1-phase cells. As shown in Fig. 1d, e, EXO1 is ubiquitinated in both G1 and G2 phases, and the ubiquitination of EXO1 in G1 cells was significantly higher compared with that in G2 cells, which explained at least one important aspect of the fluctuation in EXO1 level during the cell cycle. Considering the basal protein content of EXO1 was different between G1 and G2 cells, synchronized cell were pre-treated with proteasome inhibitor MG132 for 2 h in Co-IP experiment. The addition of MG132 in G1 cells resulted in higher amount of EXO1, while no significant changes were observed in EXO1 levels during G2 phase (Fig. 1f), which confirmed the observation that EXO1 degraded only in G1 via ubiquitin-proteasome pathway. It has been suggested that the SCF-Cyclin F (SCF) E3 ligase is responsible for the ubiquitination-mediated degradation of the EXO1 protein [16, 21], while SCF E3 ligase activity is tightly stimulated by post-translation modification of Cullin1 neddylation. As shown in Fig. 1g, increased NEDD8 interactions with Cullin1 were detected in G1 cells compared with that in G2 cells. Furthermore, the stability of EXO1 protein was significantly increased by MLN4924, an inhibitor of neddylation E1 activity (Fig. 1h).

### Increased expression of RBX1 in G1 phase prompts the degradation of EXO1 by promoting the neddylation of Cullin1 and activating SCF E3 ligase

**Fig. 2:**
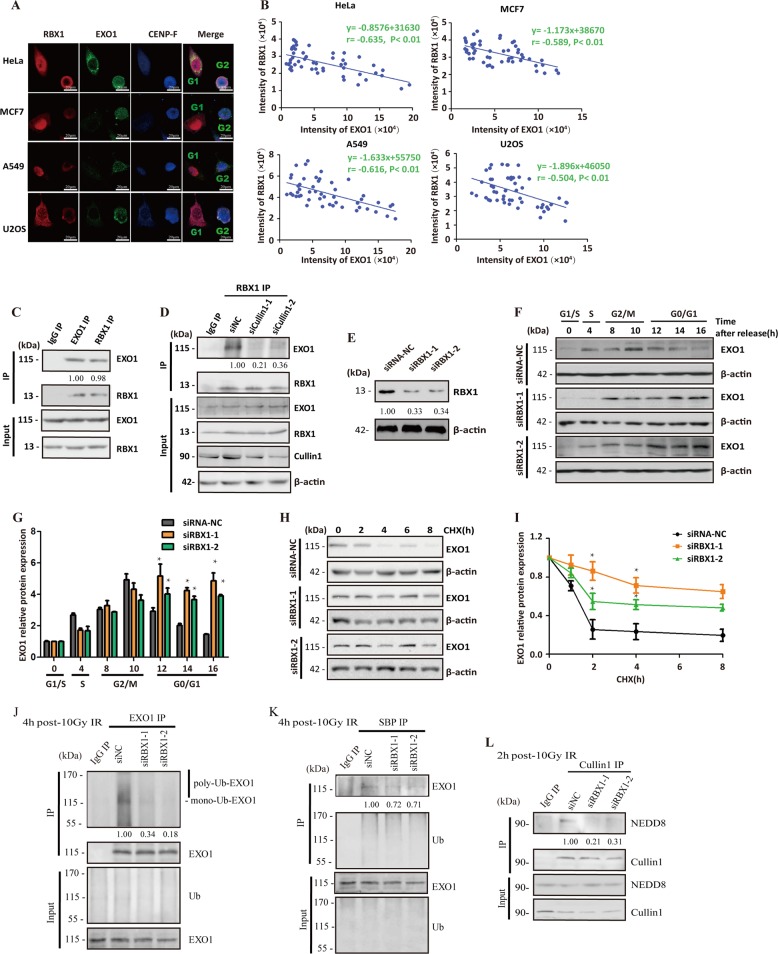
RBX1 mediates the degradation of EXO1 in G1-phase cells by promoting the neddylation of Cullin1 and activating SCF E3 ligase. **a** Immunofluorescent staining images displayed the negative correlation between RBX1 and EXO1 expression in various types of cells. HeLa, MCF7, A549, and U2OS cells were immunostained with anti-RBX1 antibody (red), anti-EXO1 antibody (green), and anti-CENP-F antibody (blue), and the images were collected under laser confocal microscopy. CENP-F staining was used to distinguish G2/M-phase cells from G1 cells. **b** Quantitative analysis of the correlation between RBX1 and EXO1 expression in multiple cell lines. The fluorescence intensities of RBX1 and EXO1 were measured using ImageJ software, and a correlation analysis was performed. *p* value less than 0.05 indicates a significant relationship between RBX1 and EXO1 expression. **c** The interaction between EXO1 and RBX1 were observed by IP-Western. **d** After knockdown of Cullin1 by siRNA in HeLa cells, the interaction between EXO1 and RBX1 were assessed. **e** Western blotting analysis verified the knockdown of RBX1 by siRNA in HeLa cells. **f** Knockdown of RBX1 augmented the EXO1 protein in G1-phase cells. HeLa cells were depleted of endogenous RBX1 using siRNA and synchronized with double-blockage of thymidine. Then, EXO1 protein levels were detected by western blotting analysis at different times after release from thymidine blockage, and the synchronized cells were monitored by flow cytometry. **g** Densitometric quantitation of western blotting analysis of EXO1 protein expression. Data are the mean ± standard deviation from three independent experiments. *p *<* 0.05 compared with the cells treated with the control siRNA-NC. **h** The effect of knocking down RBX1 on the stability of the EXO1 protein. The RBX1-knockdown cells by specific siRNA and the control cells were cultured with cycloheximide (CHX). EXO1 levels were assessed by western blotting analysis at the indicated times after CHX treatment. **i** Densitometric quantitation of western blotting analysis of EXO1 protein expression at the indicated times after CHX treatment. Data are the mean ± standard deviation from three independent experiments. *p *<* 0.05 compared with the cells treated with the control siRNA-NC. **j**, **k** Knockdown of RBX1 led to decreased ubiquitination of the EXO1 protein. HeLa cells were co-transfected with siRNA RBX1 and HA-Ub or SBP-Ub plasmid, and the immunoprecipitation (IP) product obtained with the EXO1 antibody and S-bead agarose, were subjected to western blotting analysis with the anti-flag antibody and anti-EXO1 antibody separately. **l** Knockdown of RBX1 led to decreased neddylation of Cullin1 protein. The immunoprecipitation (IP) production of Cullin1 antibody from the siRNA-mediated RBX1-knockdown HeLa cells and the control cells were subjected to western blotting analysis with anti-NEDD8 antibody

It is acknowledged that RBX1’s C-terminal RING promotes NEDD8 ligation to Cullin1 and activates SCF E3 ligase [22, 23]. However, it is not known whether this neddylation of Cullin1 is cell cycle dependent and whether associated with the cell cycle variation of EXO1 activity. Interestingly, we observed a much higher level of RBX1 protein in synchronized G1 cells compared with that in S- and G2-phase cells, while there was no difference in Cullin1 expression among different phases of the cell cycle (Fig. 1c). Therefore, we have further focused on the potential role and mechanism of RBX1 in controlling the cell cycle-associated limitation of EXO1 function. Using CENP-F as a G2/M cell marker, we observed a strongly negative correlation between the expression levels of RBX1 and EXO1 proteins in multiple cell types, including human cervical carcinoma HeLa, breast adenocarcinoma MCF7, lung carcinoma A549, and osteocarcinoma U2OS cells (Fig. 2a, b). Moreover, the interaction of RBX1 and EXO1 was demonstrated in Fig. 2c and EXO1 interacted with RBX1 in a Cullin1-dependent manner (Fig. 2d). The negative correlation between EXO1 and RBX1 expression in both G1 and G2 cells, implying that RBX1 may determine the cell phase-based choice between EXO1 function or cell cycle-dependent degradation. To examine this possibility, the effect of depleting RBX1 with siRNA (Fig. 2e) on the expression dynamics of EXO1 was assessed during the cell cycle transition. As shown in Fig. 2f, g, the decrease in EXO1 expression in G1-phase cells was tremendously mitigated by depleting RBX1. RBX1 depletion significantly increased the stability of the EXO1 protein (Fig. 2h, i) and decreased the ubiquitination of EXO1 in Co-IP experiments (Fig. 2j, k), indicating that RBX1 triggered EXO1 degradation. Moreover, the neddylation of Cullin1 was verified to be abolished after depleting RBX1 (Fig. 2l). Therefore, our results suggest that a higher level of RBX1 in the G1 phase prompted the ubiquitination-mediated degradation of EXO1 via activating SCF E3 ligase.

### RBX1 deficiency significantly sensitizes cells to irradiation

**Fig. 3:**
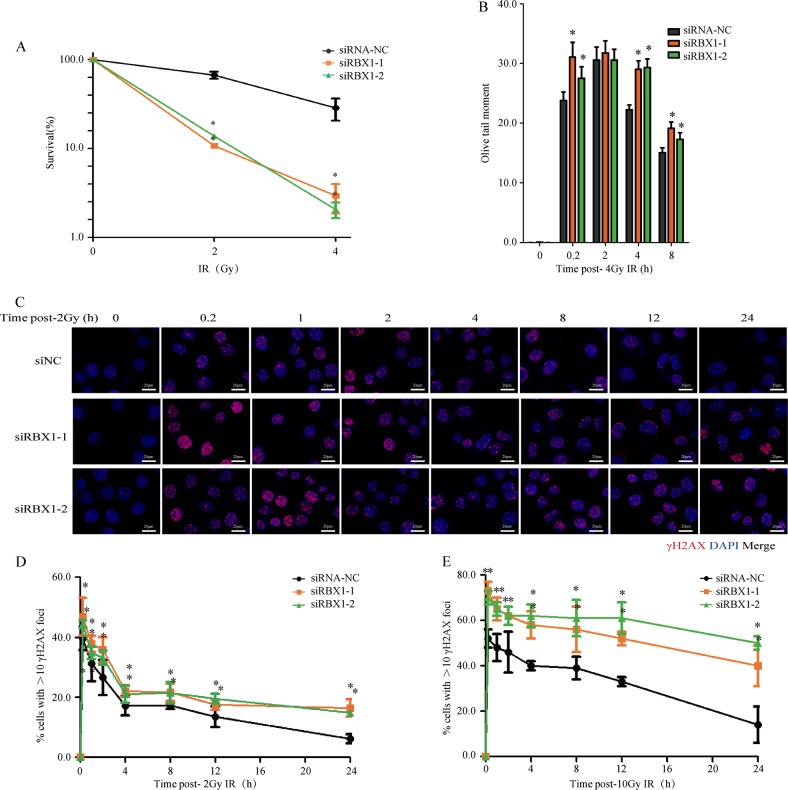
Depression of RBX1 causes radiosensitization and deficiency in DNA double-strand break repair. **a** Cell survival of HeLa cells irradiated with *γ*-rays by colony-forming ability assay. **b** Neutral comet assay of radiation-induced DNA double-strand break (DSB) repair in HeLa cells treated with RBX1 siRNA or control siRNA-NC. DNA damage was expressed as the Olive tail moment. **c** Detection of radiation-induced DNA DSBs by γH2AX foci of immunofluorescent staining in HeLa cells treated with RBX1 siRNA or control siRNA-NC. Quantitative determination of γH2AX foci as DSB indicators in RBX1-knockdown HeLa cells and control cells irradiated with 2 Gy (**d**) or 10 Gy (**e**) of *γ*-rays. In total, 50 randomly selected cells were scored in each group (mean ± standard deviation). **p *<* 0.01 compared with control siRNA-NC treated cells

The colony formation ability assay indicated that siRNA-mediated depletion of RBX1 increased the sensitivity of cells to 2 and 4 Gy IR exposure (Fig. 3a). We performed neutral comet assays to measure the resolution of DSBs, the data suggested that the repair of 4 Gy IR-induced DSBs was notably delayed in RBX1-depleted cells (Fig. 3b). Phosphorylated H2AX (γH2AX) foci were then analysed to detect the effect of depleting RBX1 on the repair capability of radiation-induced DSBs (Fig. 3c). HeLa cells were treated with specific siRNAs to knockdown RBX1, and γH2AX foci were detected in the cells at different times after exposure to 2 Gy (Fig. 3c, d) or 10 Gy (Fig. 3e). The results indicated that depletion of RBX1 caused higher levels of residual γH2AX foci, i.e., DSBs. Obviously, at 8 and 24 h after irradiation, a certain level of residual γH2AX foci still existed in cells with depleted RBX1, while very few γH2AX foci were detected in the control group. These data implicated the depression of RBX1 in the decreased efficiency of DSB repair.

### RBX1 promotes IR-induced EXO1 degradation to limit end resection in G1 phase

**Fig. 4:**
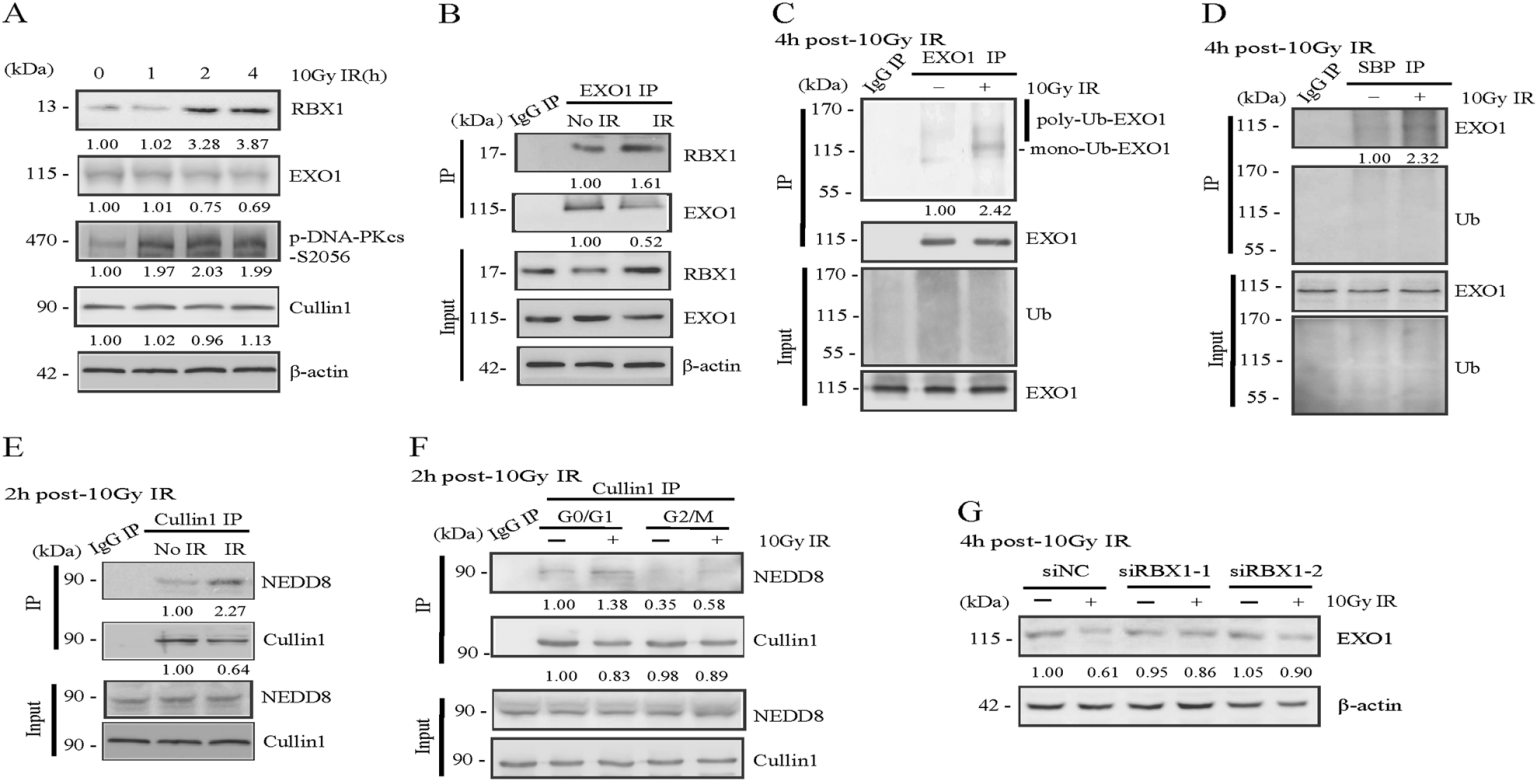
RBX1 promotes a decrease in EXO1 induced by ionizing radiation. **a** Effects of ionizing radiation on RBX1 and EXO1 protein expression. Western blotting analysis of RBX1, EXO1, Cullin1, and phosphorylated DNA-PKcs/s2056 in HeLa cells at the indicated time after exposure to 10 Gy. **b** The interaction between RBX1 and EXO1 was assessed in HeLa cells at 6 h after 10 Gy IR by immunoprecipitation assay. **c**, **d** After transfected with HA-Ub and SBP-Ub, the ubiquitination of the EXO1 protein was detected at 6 h after 10 Gy IR. To achieve the same content of EXO1 between cells treated with or without IR, proteasome inhibitor MG132 were added before ionization radiation. **e** Ionizing radiation increased the neddylation of Cullin1 protein at 2 h after 10 Gy IR. **f** HeLa cells were synchronized in G1 and G2 cells, respectively, and the neddylation of Cullin1 protein was detected at 2 h after exposed to 10 Gy IR. **g** The siRNA-NC and siRNA-RBX1 cells were exposed to 10 Gy IR and after 6 h, contents of EXO1 were evaluated with western blotting analysis

Next, we asked whether the radioprotective effect of RBX1 is relevant to its role in limiting EXO1 function in G1 cells. As shown in Fig. 4a, RBX1 expression increased significantly after irradiation. Simultaneously, EXO1 significantly decreased, and phosphorylated DNA-PKcs pS2056 also increased, while there was no change in the Cullin1 protein level. The interaction between RBX1 and EXO1 increased at 6 h after 10 Gy IR (Fig. 4b). Meanwhile, the ubiquitination of EXO1 (Fig. 4c, d) and neddylation of Cullin1 (Fig. 4e) increased in the irradiated HeLa cells. Importantly, increased neddylation of Cullin1 was detected at 1 h post-irradiation in synchronized G1 cells but not in G2 cells (Fig. 4f). Knockdown of RBX1 attenuated the decrease in EXO1 induced by irradiation (Fig. 4g).

**Fig. 5:**
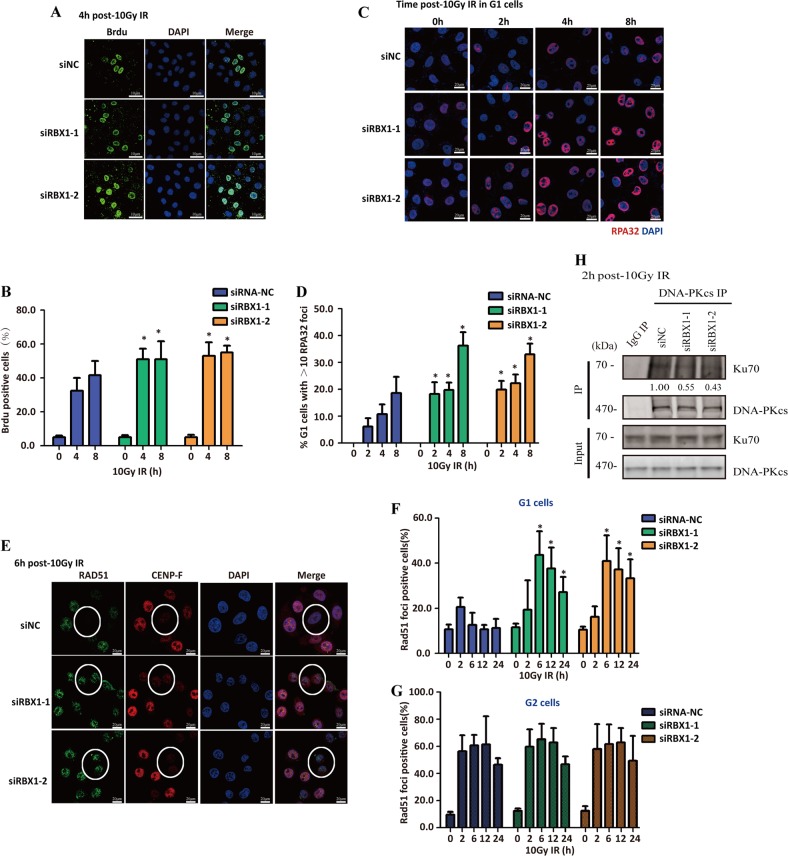
Knockdown of RBX1 leads to increased end resection and RAD51 foci formation in G1-phase cells. **a** Knockdown of RBX1 increased end resection of HeLa cells at 4 h after 10 Gy IR as displayed by the immunostaining with anti-BrdU antibody. Representative images of 10 Gy-irradiated HeLa cells immunostained with anti-BrdU antibody. **b** Quantitative determination of BrdU foci observation. BrdU staining >20 dots in the nucleus was identified as positive cell. *p *<* 0.05 compared with control siRNA-NC treated cells at the same time point after irradiation. **c** After synchronized at G1-phase, cells depleted with siRBX1 or siNC were exposed to 10 Gy, and RPA32 foci positive cells were observed at different time point. Representative images of 10 Gy-irradiated synchronized G1 HeLa cells immunostained with anti-RPA32 antibody. (**d**) Quantitative determination of RPA32 foci. *p *<* 0.05 compared with control siRNA-NC treated cells at the same time point after irradiation. **e** Knockdown of RBX1 increased RAD51 foci formation in G1-phase cells. CENP-F was used to distinguish G2 cells from G1 cells. The cells marked with red circles represent G1 cells with lower CENP-F expression levels. siNC represents HeLa cells treated with non-specific siRNA as a control. Quantitative determination of RAD51 foci as an HR pathway indicator in RBX1-knockdown HeLa cells and control cells at 6 h after 10 Gy irradiation in G1-phase (**f**) and G2-phase cells (**g**). *p *<* 0.05 compared with control siRNA-NC treated cells at the same time point after irradiation. **h** Knockdown of RBX1 decreased the interaction between DNA-PKcs and Ku70. SiRNA-mediated RBX1-knockdown HeLa cells and the control cells were synchronized in G1 cells. The immunoprecipitation (IP) production of DNA-PKcs antibody were subjected to western blotting analysis with anti-Ku70 antibody

To illustrate how cells would benefit from EXO1 degradation after IR exposure, we further investigated the effects of RBX1 on end resection and HR repair in G1-phase cells by monitoring ssDNA formation and RAD51 foci. DNA end resection results in the generation of ssDNA ends, which can be monitored by the immunofluorescent staining of anti-BrdU antibody binding and Replication Protein A32(RPA32) [24]. The results showed that knockdown of RBX1 increased the foci formation of anti-BrdU antibody binding (Fig. 5a, b) and RPA32 (Fig. 5c, d), suggesting that RBX1 can restrain DNA end resection of DSBs in G1 phase. Besides, after 4 h post-10 Gy IR, synchronized cells were arrested in G1 phase, indicating the increase of RPA32 foci was due to knockdown of RBX1 other than the change of cell cycle (Fig. S1). CENP-F was used as a G2/M marker to distinguish G1 cells (Fig. 5e), and depletion of RBX1 significantly increased RAD51 foci mainly in G1-phase cells with lower levels of CENP-F expression (Fig. 5e, f) but not in G2 cells (Fig. 5e, g). Furthermore, knockdown of RBX1 diminished the interaction between DNA-PKcs and Ku70, which supported RBX1 promote NHEJ repair in G1 cells through limiting DNA end resection (Fig. 5h).

### EXO1 depletion mitigated DNA repair defects due to RBX1 reduction

**Fig. 6:**
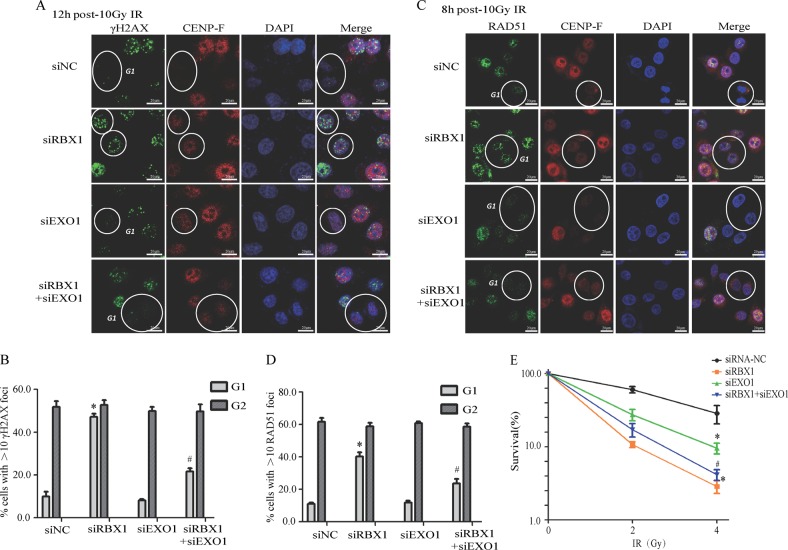
EXO1 depletion mitigated DNA repair defects due to RBX1 reduction. **a**, **b** HeLa cells transfected with siRNA-RBX1 or siRNA-NC were co-cultured with 10 μM BrdU for 16 h, then cells were exposed to 10 Gy irradiation, residual γH2AX foci were observed with CENP-F as a marker to distinguish G1 and G2 cells. The percentages of HeLa cells with >10 γH2AX foci were counted. In total, 50 randomly selected cells were scored in each group. *p *<* 0.01 compared with control siRNA-NC treated cells. ^#^*p* *<* 0.01 compared with control siRNA-RBX1 treated cells. **c**, **d** At 8 h after siRNA-RBX1 or siRNA-NC cells exposed to 10 Gy IR, cells were immunostained with anti-RAD51 antibody. Quantitative determination of RAD51 foci positive cells and 50 randomly selected cells were scored in each group. *p *<* 0.01 compared with control siRNA-NC treated cells. ^#^*p* < 0.01 compared with control siRNA-RBX1 treated cells. **e** Cell survival of HeLa cells irradiated with *γ*-rays by colony-forming ability assay to evaluate the combination effects of RBX1 and EXO1 compared with RBX1 alone on radiosensitivity

Furthermore, we questioned whether ubiquitination degradation of EXO1 was the immediate cause for the radioresistance effects of RBX1. At 12 h after 10 Gy irradiation, a certain level of residual γH2AX foci still existed in cells with depleted RBX1, while very few γH2AX foci were detected in the control group. The RBX1-depleted cells with positive γH2AX foci was smaller than that in siRNA-RBX1 group. Inactivation of EXO1 partially reduces gamma-H2AX foci in cells depleted for RBX1 (Fig. 6a, b). We also observed RAD51 foci as the indicator of DSB repair. Knockdown of RBX1 increased RAD51 foci formation in G1-phase cells, and EXO1 siRNA reversed the increased Rad51 foci generation in G1 cells (Fig. 6c, d). The colony formation ability assay indicated that the depletion of EXO1 mitigated the augment of radiosensitivity of cells due to lack of RBX1(Fig. 6e). Therefore, the combination knockdown of RBX1 and EXO1 rescued the inhibitory effects of RBX1 on DSB repair, and RBX1 did not disturb γH2AX foci and RAD51 foci in G2 cells, which suggested EXO1 was responsible for the interference of RBX1 on DSB repair, especially in G1 cells.

### Involvement of DNA-PKcs in the pathway regulation of RBX1-mediated EXO1 degradation in G1 phase

**Fig. 7:**
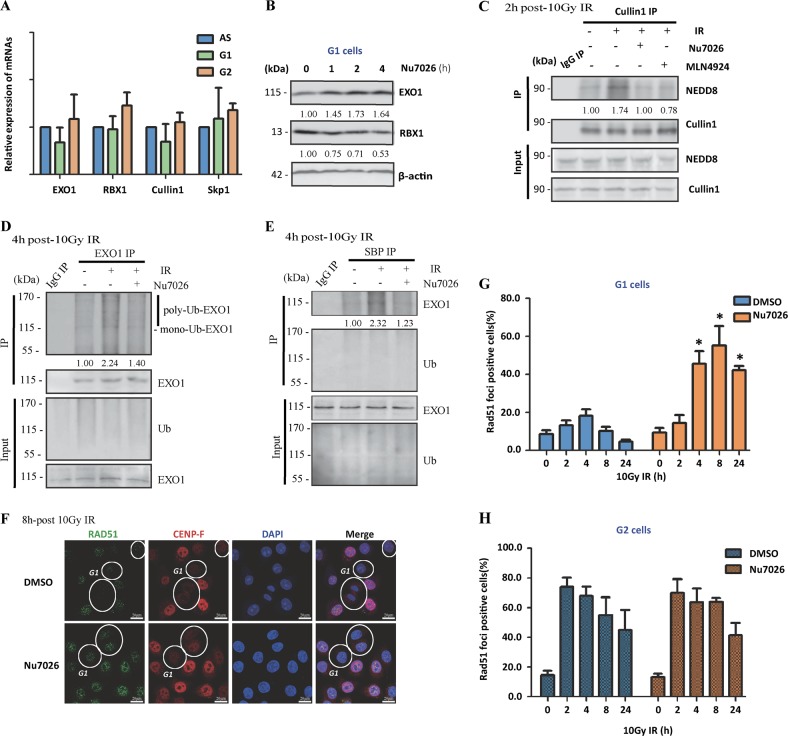
Inactivation of DNA-PKcs increased EXO1 protein expression and RAD51 foci formation in G1-phase cells through depression of RBX1 protein. **a** The mRNA expression of EXO1, RBX1, Cullin1 and Skp1 in different phases of HeLa cells was quantified by RT-PCR. **b** The DNA-PKcs inhibitor Nu7026 (10 μM) increased EXO1 expression and decreased RBX1 expression in G1-phase HeLa cells. **c** Nu7026 and the neddylation inhibitor MLN4924 attenuated the increase in Cullin1 neddylation at 2 h after 10 Gy irradiation. **d**, **e** Nu7026 attenuated the increased ubiquitination of EXO1 induced by 10 Gy irradiation. After HeLa were transfected with HA-Ub and SBP-Ub plasmid for 48 h, the immunoprecipitation (IP) product obtained with the EXO1 antibody and S-bead agarose, were subjected to western blotting analysis with the anti-flag antibody and anti-EXO1 antibody separately. To achieve the same content of EXO1 for immunoprecipitation, MG132 were added before ionization radiation. **f** Representative images displayed the effect of Nu7026 on RAD51 foci formation in 10 Gy-irradiated HeLa cells. After 8 h, cells were immunostained with anti-RAD51 antibody and marked with red circles represent G1 cells with lower CENP-F expression levels. **g**, **h** Quantitative determination of RAD51 foci in G1-phase and G2-phase cells. *p *<* 0.05 compared with control DMSO-treated cells at the same time point after the treatment

We next explored the reason for the higher expression of RBX1 in G1 phase than in G2 phase. First, we examined potential variation in transcriptional levels during the cell cycle, and there were no significant differences in the mRNA expression of EXO1, RBX1, Cullin1, or Skp1 among different phases of the cell cycle (Fig. 7a). As mentioned above, increased phosphorylated DNA-PKcs S2056 was found in G1-phase cells (Fig. 1c), which was further induced by irradiation (Fig. 4a) along with the characteristic pattern of RBX1 expression. DNA-PKcs are activated in G1 phase to execute NHEJ repair. Then, we checked whether DNA-PKcs regulates the protein level of RBX1. Obviously, the DNA-PKcs inhibitor Nu7026 diminished RBX1 and increased EXO1 in synchronized G1-phase HeLa cells (Fig. 7b), suggesting that DNA-PKcs are responsible for the increase in RBX1. Furthermore, we verified whether DNA-PKcs regulated the neddylation of Cullin1 and ubiquitination of EXO1. The results demonstrated that the increased neddylation of Cullin1 induced by irradiation was diminished by both Nu7026 and the neddylation inhibitor MLN4924 (Fig. 7c). The radiation-induced ubiquitination of EXO1 was also attenuated by Nu7026 (Fig. 7d, e). Importantly, we found that Nu7026 resulted in a significant increase in RAD51 foci, a critical component of the HR pathway, specifically in the G1 phase of irradiated cells (Fig. 7f, g) but not in the G2 phase (Fig. 7f, h). These data suggest that in the G1 phase, a higher level of RBX1 is maintained by the activated DNA-PKcs, which further prompts SCF E3 activity and promotes EXO1 degradation, consequently restraining the role of the HR pathway.

**Fig. 8:**
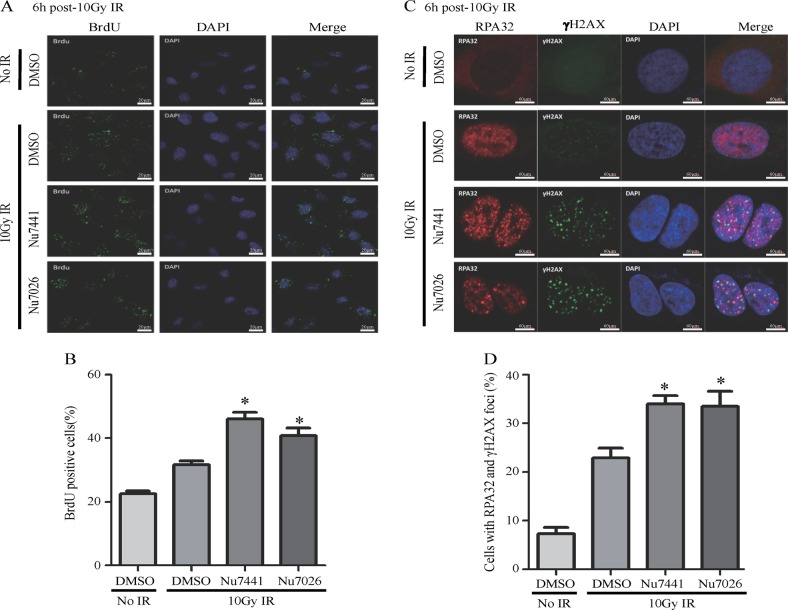
Inactivation of DNA-PKcs increases the ssDNA generation of DSB ends. **a** The production of ssDNA was visualized by immunostaining with anti-BrdU antibody. HeLa cells were pre-treated with 10 μM BrdU for 16 h, then cells were collected at 6 h after 10 Gy irradiation. The DNA-PKcs inhibitors Nu7441 and Nu7026 increased BrdU foci and ssDNA generation. **b** Quantitative determination of BrdU foci in 10 Gy-irradiated cells treated with or without Nu7441 or Nu7026. *p *<* 0.05. **c** The production of ssDNA was visualized by immunostaining with RPA32 antibody. The DNA-PKcs inhibitors Nu7441 and Nu7026 increased RPA32 foci and ssDNA generation. **d** Quantitative determination of RPA32/γH2AX double-labeled foci positive cells in 10 Gy-irradiated cells treated with or without Nu7441 or Nu7026. *p *<* 0.05. More than 50 cells were scored in each experiment

The role of DNA-PKcs in DSB end resection was further investigated by monitoring ssDNA formation by immunofluorescence staining with BrdU and RPA32 antibodies in cells treated with the DNA-PKcs inhibitors Nu7441 or Nu7026. The results indicated that the inactivation of DNA-PKcs significantly increased BrdU binding foci compared with that in the cells treated with DMSO (Fig. 8a, b). We also explored whether DNA-PK inhibitors alters the accumulation of RPA32 foci on chromatin after DNA damage. The results indicated that Nu7441 or Nu7026 augmented the numbers of RPA32 and γH2AX co-foci (Fig. 8c, d), demonstrating that activated DNA-PK limited the end resection of DSBs.

## Discussion

**Fig. 9:**
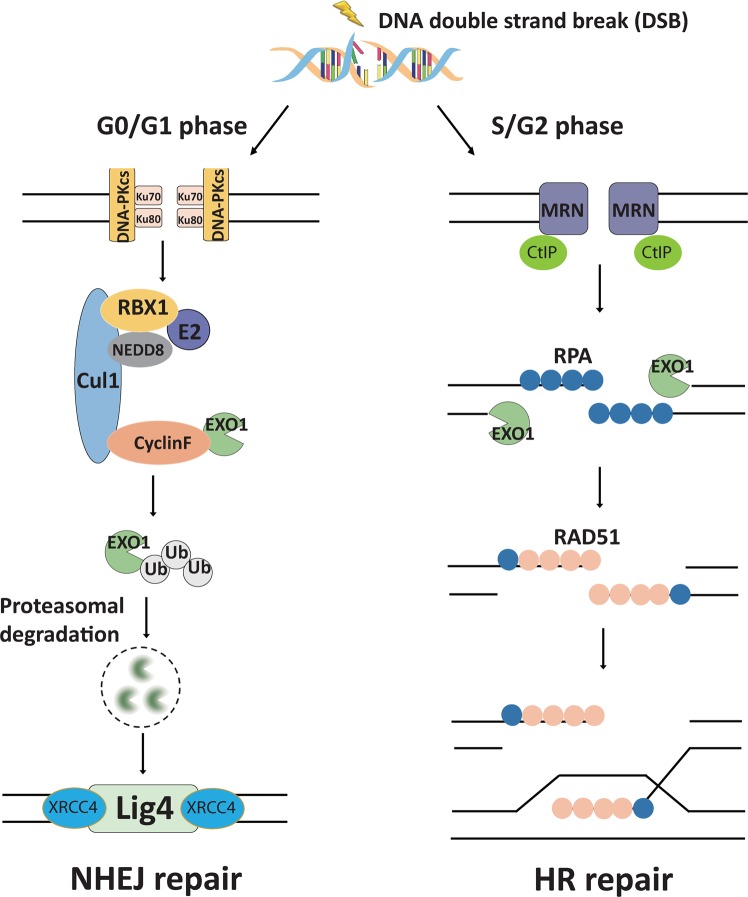
Model summarizing the mechanism of DNA-PK/RBX1/EXO1 pathway on limiting end resection in G1 phase

EXO1 is a critical component in the end resection of the HR pathway of DNA DSB repair. It has been demonstrated that CDK1/2-mediated phosphorylation of EXO1 at four C-terminal S/TP sites regulates the repair pathway choice during S/G2 phases of the cell cycle [25]. And HR activity is intensely limited in the G1 phase. Currently, we have found that the EXO1 protein was extremely suppressed during the G1 phase through ubiquitin proteasome pathway and we further observed a much higher level of RING-box protein 1 (RBX1) protein in the G1 phase than in the S/G2 phases. Importantly, this cell cycle-associated expression pattern of RBX1 protein was negatively correlated with EXO1 protein expression. Moreover, knockdown of RBX1 reversed the degradation of EXO1 in G1 cells. Finally, we revealed that RBX1-mediated degradation of the EXO1 protein contributes to the choice of the NHEJ pathway of DNA DSB repair in the G1 phase (Fig. 9).

It has been demonstrated that K796 of EXO1 is a target of Skp-Cullin-F-box (SCF) E3 ubiquitin ligase in response to DNA damage, and Cullin1, rather than Cullin 2–5 of the Cullin family, is responsible for EXO1 degradation [21]. Cullin1 recruits the F-box family of substrate specificity factors and binds the RING domain protein RBX1 to subsequently recruit E2 [26, 27]. RBX1 was identified as an essential protein for cell survival through activating ubiquitination modification of target protein, but its effects on DSB repair was not explored before [28].

EXO1-dependent DSB end resection is a critical determining factor for G1-phase cells to preferably select the NHEJ rather than the HR pathway of DNA DSB repair. We observed a much higher level of RBX1 protein in the G1 phase than in the S/G2 phases. Importantly, this cell cycle-associated expression pattern of RBX1 protein was negatively correlated with EXO1 protein expression. Moreover, knockdown of RBX1 reversed the degradation of EXO1 in G1 cells. Considering that the ubiquitination and degradation of EXO1 is mediated by the SCF E3 ligase, and the level of Cullin1 protein is constant during the cell cycle, RBX1 could be a key regulator of the expression of EXO1 during the cell cycle.

The activity of SCF ligase is stimulated by covalent modification of Cullins with the ubiquitin-like protein NEDD8, which is mediated by RBX1 [29–31]. RBX1 promotes NEDD8 ligation to lys720 in Cullin1’s C-terminal domain and is essential for Cullin1 neddylation and SCF activity [22]. Our results suggested that RBX1 deficiency resulted in a decline in EXO1 ubiquitination and an increase in EXO1 stability through diminishing neddylation of Cullin1. Importantly, knocking down RBX1 significantly activated the end resection of DNA DSBs in irradiated G1 cells but not in G2 cells, which was shown as increased formation of ssDNA.

RBX1 was reported to be over-expressed in human tumor tissues, and was suggested to contribute to tumor progression and poor prognosis by regulating cell proliferation, apoptosis and senescence [32]. RBX1 silencing causes the accumulation of DNA replication licencing proteins CDT1 and ORC1, and leads to DSBs, DNA damage response and increased radiosensitivity [33]. Our study demonstrated that the expression of RBX1 protein was increased by ionizing radiation, and RBX1 silencing led to deficiency of DSB repair.

Although HR repair occurs mainly in later S phase and the G2/M phase, HR can also be activated in G1 phase [34]. Increased EXO1 resection activity leads to the decreased association of the Ku complex with DSBs and enhanced DSB resection in G1, indicating that EXO1 has a direct function in preventing Ku association with DSBs and end resection in G1 [18]. The decrease of interaction between DNA-PKcs and Ku70 was observed in irradiated-siRBX1 cells, compared with control cells group. Our results indicated that HeLa cells depleted of RBX1 gave rise to the activation of DNA end resection and HR in G1 phase. After irradiation, increased RBX1 expression was tightly associated with a decrease of EXO1 levels and restriction of DSB end resection in the G1 phase, guaranteeing proper choice of the NHEJ repair pathway and maintenance of genomic stability.

Based on the existing reports, RBX1 has complex impacts on regulating protein degradation with DSB sites by activating SCF E3 ligase [35]. Cullin1^Skp2^ E3 ligase is a critical component to facilitate ATM recruitment to the DSB sites for activation and promote NHEJ repair by mediating NBS1 ubiquitination [36]. PALBs ubiquitylation by Cullin3 E3 ligase suppresses its interaction with BRCA1 in order not to ignite HR repair in G1 cells [34]. In our study, knockdown of both RBX1 and EXO1 rescued the increase of γH2AX foci and RAD51 foci in G1 cells and radiosensitivity by depletion of RBX1 alone. It is reasonable to deduce that, although the effects of RBX1 on CtIP and NBS1 have not been investigated, the RBX1-Cullin pathway is one of the important mechanisms that modulates EXO1 activity and the choice of DSB repair system during the cell cycle.

It has been reported that cells depleted of DNA-PKcs or treated with DNA-PK inhibitor induced IR-induced RAD51 foci formation [37]. We found that the effects of a DNA-PK inhibitor on RAD51 nucleofilament formation and HR function mainly occurred in G1-phase cells, while no obvious changes occurred in G2 cells. DNA-PKcs promotes the ubiquitination of EXO1 in the G1 phase by increasing RBX1 expression and activation of SCF E3 ligase. However, the more precise mechanism by which DNA-PKcs regulates RBX1 expression is worthy of exploring in the future.

In summary, our results indicate that the EXO1 is inactivated in the G1 phase by SCF E3 ligase-mediated ubiquitination and degradation. RBX1 is the major regulator of this cell cycle-dependent degradation of EXO1 and consequently remarkably limits end resection, the formation of single-strand DNA ends and HR activity in the G1 phase but not in the S and G2 phases. In addition, DNA-PK activity is responsible for increased RBX1 protein expression, limiting the formation of DSB end ssDNA and suppressing the HR repair pathway in G1 cells. This study provides a new cell cycle-associated mechanism for the choice of DNA DSB repair pathway.

## Supplementary information

Figure S1 Legand

Figure S1
