# Supplementary material for: RBX1 prompts degradation of EXO1 to limit the homologous recombination pathway of DNA double-strand break repair in G1 phase
Source: Cell Death Differ. 2019 Sep 27;27(4):1383–97. doi: 10.1038/s41418-019-0424-4 (PMC7205894; doi:10.1038/s41418-019-0424-4)
Supplement: Supplementary file 1 — Figure S1 Legand [file 41418_2019_424_MOESM1_ESM.docx]

**Figure S1. Cell cycle analysis of G1 cells after different exposure times to 10Gy IR.**

HeLa cells were synchronized in G1 phases by double-blockage of thymidine, and then were collected in different times after exposed to 10Gy IR. The cell cycle was analysed by flow cytometry analysis.
